# In Situ Thermal Decomposition of Potassium Borohydride for Borophene Synthesis and Its Application in a High-Performance Non-Volatile Memory Device

**DOI:** 10.3390/nano15050362

**Published:** 2025-02-26

**Authors:** Qian Tian, Xinchao Liang, Maoping Xu, Yi Liu, Qilong Wu, Guoan Tai

**Affiliations:** State Key Laboratory of Mechanics and Control for Aerospace Structures, Laboratory of Intelligent Nano Materials and Devices of Ministry of Education, College of Aerospace Engineering, Nanjing University of Aeronautics and Astronautics, Nanjing 210016, China; sx2201054@nuaa.edu.cn (Q.T.); liangxinchao@nuaa.edu.cn (X.L.); mpx_ins@nuaa.edu.cn (M.X.); liuyi061@nuaa.edu.cn (Y.L.); sz2201086@nuaa.edu.cn (Q.W.)

**Keywords:** borophene, two-dimensional material, chemical vapor deposition, semiconductor, non-volatile memory device

## Abstract

Borophene, a revolutionary two-dimensional (2D) material with exceptional electrical, physical, and chemical properties, holds great promise for high-performance, highly integrated information storage systems. However, its metallic nature and structural instability have significantly limited its practical applications. To address these challenges, hydrogenated borophene has emerged as an ideal alternative, offering enhanced stability and semiconducting properties. In this study, we report a novel and scalable method for synthesizing hydrogenated borophene via the in situ thermal decomposition of potassium borohydride in a substrate-free environment. This approach enables the production of borophene with outstanding crystallinity, uniformity, and continuity, representing a significant advancement in borophene fabrication techniques. Furthermore, the hydrogenated borophene-based non-volatile memory device we developed exhibits a high ON/OFF-current ratio exceeding 10^5^, a low operating voltage of 2 V, and excellent long-term cycling stability. These groundbreaking results demonstrate the immense potential of 2D borophene-based materials in next-generation high-performance information storage devices.

## 1. Introduction

Since the exfoliation of graphene [1], two-dimensional (2D) materials have captured significant attention in materials science due to their unique structures and superior properties. The research on graphene [2], transition metal dichalcogenides (TMDs) [3], black phosphorus (BP) [4], and other 2D materials has driven technological advancements in fields such as electrochemistry, energy storage, memory devices, and sensor technologies. However, these traditional 2D materials face limitations in practical applications, such as narrow bandgaps, poor chemical stability, and limited tunability, necessitating the development of novel 2D materials to overcome these challenges.

Borophene has emerged as a research hotspot due to its exceptional physical and chemical properties, including high mechanical strength, flexibility, superior electron mobility, optical transparency, and excellent thermal conductivity [5,6,7]. Since its first successful synthesis on copper foil [8], borophene has significantly advanced the frontier of materials science in less than a decade. Notably, borophene exhibits multiple 2D allotropes, each with tunable and diverse electronic band structures. For instance, the intrinsic α-phase borophene exhibits an indirect bandgap, while structures such as δ_6_, β_12_, χ_3_, and δ_3_ primarily display metallic properties [9,10,11].

To regulate borophene’s electronic properties effectively, researchers have explored three primary strategies: introducing defects, cutting borophene into nanoribbons, and surface functionalization. For example, introducing vacancies and strain into the δ_6_ structure can expand the bandgap to 0.63 eV; cutting borophene into nanoribbons achieves a bandgap of 0.26 eV; and functionalizing the nanoribbons’ surfaces with hydrogen atoms further increases the bandgap to 1.07 eV. However, while introducing defects or cutting into nanoribbons can modulate the bandgap, these approaches often significantly reduce carrier mobility due to defect sites or edge scattering. In contrast, surface functionalization emerges as a more ideal strategy, offering both bandgap tunability and high carrier mobility [6,12,13]. These bandgap regulation methods have greatly expanded borophene’s applications in electronics, biomedicine, and energy storage [14,15,16,17]. Particularly, hydrogenated borophene inherits borophene’s excellent electronic properties while dramatically improving its stability through hydrogen functionalization [18,19], paving the way for high-performance, low-power electronic devices.

In this study, we successfully synthesized α’-4H-borophene with a semiconducting phase in a substrate-free environment. Potassium borohydride (KBH_4_) was employed as the borophene source, using metal-catalyzed in situ thermal decomposition under low-pressure, hydrogen-rich conditions. Scanning electron microscopy (SEM) and atomic force microscopy (AFM) revealed excellent flatness and continuity of the nanosheets, with an average thickness of approximately 3.7 nm. Transmission electron microscopy (TEM) and selected area electron diffraction (SAED) confirmed the nanosheets’ outstanding crystallinity and structural homogeneity, consistent with previously reported α’-4H-borophene [20]. Additionally, Raman spectroscopy, photoluminescence (PL), and X-ray photoelectron spectroscopy (XPS) analyses verified the elemental composition, bonding configurations, and semiconducting properties, aligning with theoretical predictions. Furthermore, we developed a borophene-based non-volatile memory device, utilizing α’-4H-borophene as the charge-trapping material and polyvinylpyrrolidone (PVP) as the insulating layer. The device demonstrated exceptional performance, achieving a high ON/OFF-current ratio exceeding 10^5^, a low operating voltage of 2 V, and excellent cycling durability and long-term stability. These findings highlight the superior potential of hydrogenated borophene in electronic devices and firmly establish a foundation for its use in high-performance, highly integrated information storage systems.

## 2. Materials and Methods

### 2.1. Materials Synthesis

All raw materials were used as received without additional treatment. KBH_4_ (99.9%) and PVP (high-purity K30) were obtained from Shanghai Aladdin Biochemical Science and Technology Co., Ltd. (Shanghai, China). Glass slides were purchased from Jingshengda Science and Technology Co., Ltd. (Chengdu, China). Ethanol (99.7%), isopropanol (99.7%), and acetone (99.7%) were supplied by Sinopharm Chemical Reagent Co. (Shanghai, China). High-purity hydrogen gas (99.999%) was sourced from Nanjing Wenda Special Gas Co., Ltd. (Nanjing, China), and ultrapure water with a resistivity of 18.25 MΩ·cm was prepared using the Milli-Q Reference system (Darmstadt, Germany).

**Figure 1:**
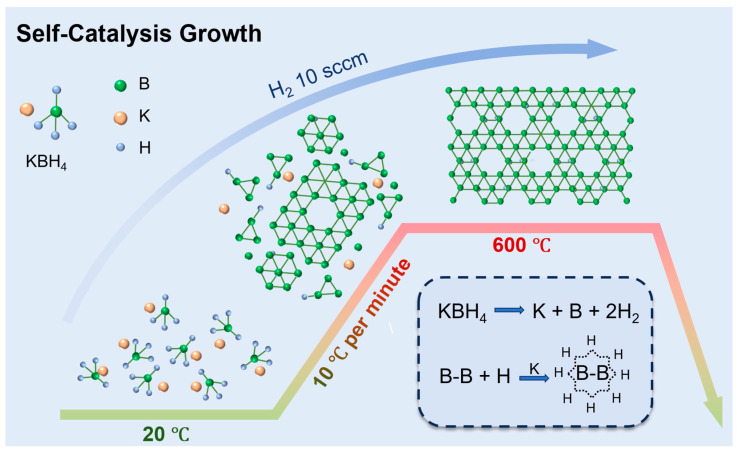
Schematic diagram of in situ thermal decomposition of KBH_4_.

Borophene nanosheets were synthesized using a chemical vapor deposition (CVD) system, with the experimental setup illustrated in Figure S1a. The synthesis of hydrogenated borophene was achieved through the in situ thermal decomposition of KBH_4_ powder, as shown in Figure 1. Initially, 500 mg of KBH_4_ powder was weighed inside a nitrogen-filled glove box, thoroughly ground, and placed in a quartz boat with an inner diameter of approximately 25 mm. The boat was positioned at the center of the T_1_ zone in the tube furnace. After sealing the furnace tube, a mechanical pump was used to evacuate air and moisture until the pressure dropped below 0.1 Pa. Subsequently, 10 sccm of high-purity H_2_ was introduced to purge the system for 10 min. The pressure was then stabilized at 50 Pa by adjusting the flange at the pump’s outlet.

The KBH_4_ powder was gradually heated from room temperature to 600 °C at a ramp rate of 10 °C/min and maintained at this temperature for 2 h. The heating curve is provided in Figure S1b. During this process, KBH_4_ underwent thermal decomposition, yielding borophene nanosheets. Following growth, a portion of the sample was dissolved in 10 mL of deionized water and transferred to a centrifuge tube. Ultrasonication was performed for 15 min to disperse the material. The dispersion was then centrifuged at 10,000 rpm for 15 min, and the supernatant was discarded. Fresh deionized water was added, and the washing process was repeated five times to remove impurities. Finally, the purified dispersion was dried in a vacuum oven at 60 °C for 12 h, producing pure borophene powder.

### 2.2. Materials Characterization

The morphology of the borophene nanosheets was characterized using a scanning electron microscope (SEM, Nova Nano SEM 450, FEI, Hillsboro, OR, USA) and a transmission electron microscope (TEM, Talos F200S, Thermo Scientific, Waltham, MA, USA). Selected area electron diffraction (SAED) patterns were obtained using the FEI Talos F200S with an accelerating voltage of 200 kV. The thickness of the synthesized borophene nanosheets was analyzed by atomic force microscopy (AFM) in tapping mode using a Smart SPM system (HORIBA Scientific, Kyoto, Japan). Raman and photoluminescence (PL) spectroscopy measurements were conducted at room temperature using a LabRAM HR Evolution spectrometer (HORIBA Jobin Yvon, Montpellier, France) with a 325 nm He-Cd laser. Ultraviolet-visible (UV–Vis) absorption spectra of the borophene nanosheets were recorded with a UV-3600 spectrophotometer (Shimadzu, Kyoto, Japan). The surface chemistry and elemental composition of the materials were analyzed by X-ray photoelectron spectroscopy (XPS) using a ESCALAB 250Xi spectrometer (Thermo Scientific, Waltham, MA, USA) equipped with a monochromatic Al Kα source (*hν* = 1486.65 eV). The measurements were performed in an ultrahigh vacuum chamber with pressures as low as 1.0 × 10^−9^ Pa, and the spectra were processed using Avantage software (v6.6.0) with an energy step of 0.05 eV and a pass energy of 30 eV.

### 2.3. Fabrication and Measurement of Memory Device

Glass slides were first cut to dimensions of 25 × 25 mm^2^ and cleaned ultrasonically in sequence with a glass cleaning solution, acetone, isopropanol, and ethanol, each for 15 min. The slides were then dried using nitrogen gas. For the bottom electrodes, 100 nm-thick gold interdigitated electrodes were sputtered onto the slides in a vacuum sputter coater using a custom-designed mask. Subsequently, the electrodes underwent surface treatment in an oxygen plasma cleaner to enhance hydrophilicity. To prepare the active layer, borophene nanosheets were first dispersed in ethanol via ultrasonication. This dispersion was then mixed with a polyvinylpyrrolidone (PVP) solution and ultrasonicated again to achieve a homogeneous mixture. Any aggregates formed during this process were removed by centrifugation. The resulting mixture was spin-coated onto the gold electrode surface and dried at 80 °C to form the active layer. Finally, the top electrode was fabricated by sputtering 100 nm-thick silver interdigitated electrodes using the same sputtering method. The two sets of electrodes were aligned perpendicularly to each other, as illustrated in Figure S2. Performance testing of the borophene-based non-volatile memory device was carried out using a four-probe station. Positive and negative probes were connected to the gold and silver electrodes, respectively. Electrical measurements were conducted using a Keithley 2450 source meter (Keithley Instruments, Cleveland, OH, USA) under ambient temperature and atmospheric pressure.

## 3. Results and Discussion

**Figure 2:**
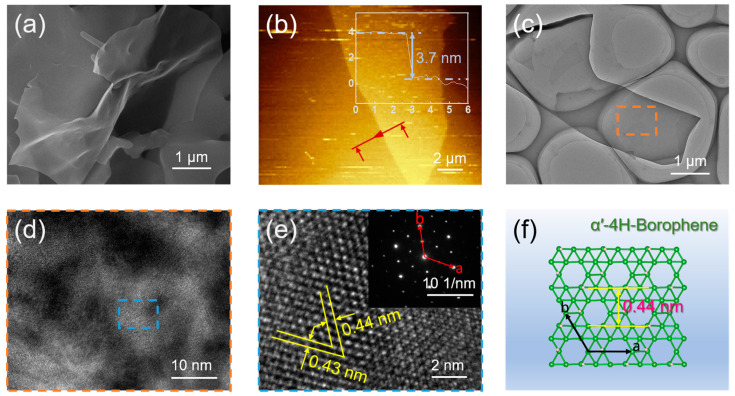
Morphological and structural characterization of borophene nanosheets: (**a**) SEM image of borophene nanosheets; (**b**) AFM image of borophene nanosheets; (**c**) Low-resolution TEM image of borophene nanosheets; (**d**) Enlarged HRTEM image from the orange marked area in (**c**); (**e**) HRTEM image from the blue marked area in (**d**); The corresponding SAED image shown in the inset; (**f**) Theoretical structural model of α’-4H-borophene.

To examine the morphology and structure of the synthesized samples, the purified borophene dispersion was deposited onto SiO_2_/Si substrates and microgrid copper grids for characterization. SEM images of the borophene nanosheets on SiO_2_/Si substrates revealed their ultrathin thickness, excellent continuity, and uniformity (Figure 2a). Additional SEM images at various growth temperatures (Figure S3) indicated that low-temperature growth resulted in ruptured and inhomogeneous nanosheets. As the temperature increased, nanotubular structures began to form on the surface, suggesting that 600 °C was the optimal temperature for growing high-quality nanosheets. AFM images further confirmed the superior quality of the synthesized nanosheets (Figure 2b), showing a uniform thickness of approximately 3.7 nm, consistent with multilayered borophene nanosheets. TEM imaging provided additional insights, revealing the clean and ultrathin structure of the nanosheets (Figure 2c). A high-resolution transmission electron microscopy (HRTEM) image from the orange rectangular region in Figure 2c is shown in Figure 2d. It highlights the nanosheets’ excellent crystallinity and homogeneity at the micrometer scale. Further magnification of the blue rectangular region in Figure 2d produced the HRTEM image in Figure 2e, displaying a well-ordered crystalline structure with measured lattice spacings of a = 0.43 nm and b = 0.44 nm, and an interplanar angle of approximately 60°. The selected area electron diffraction (SAED) pattern, shown in the inset of Figure 2e, corroborates these findings, confirming that the synthesized borophene aligns perfectly with the theoretically predicted α’-4H-borophene structure, as depicted in Figure 2f [20].

**Figure 3:**
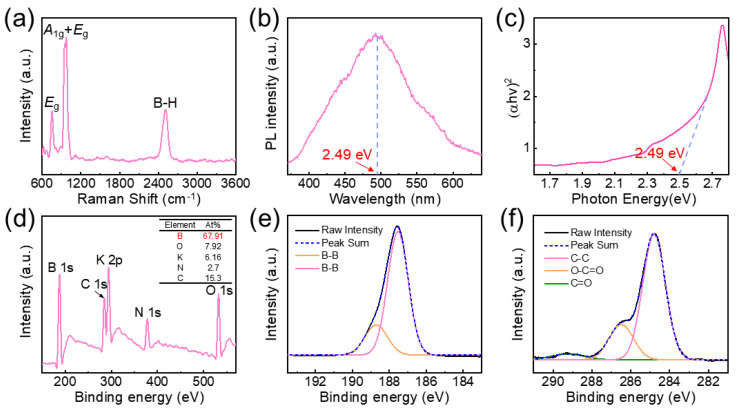
Characterization of optical and elemental properties of borophene nanosheets: (**a**) Raman plot; (**b**) Photoluminescence spectra of borophene nanosheets; (**c**) UV–Vis absorption spectra; (**d**) Full XPS spectra; (**e**) XPS spectrum of B1s; (**f**) XPS spectrum of C1s.

To investigate the potential applications of the borophene nanosheets, their optical and electrical properties were thoroughly characterized. Figure 3a shows the Raman spectra of the borophene samples, revealing three distinct Raman peaks at 746 cm^−1^ (*E*_g_ mode), 1080 cm^−1^ (*A*_1g_ + *E*_g_ mode), and 2500 cm^−1^ (B-H peak) [21,22]. The B-H peak is attributed to the formation of hydrogen-terminated bonds at the edges of the borophene during its growth in a hydrogen-rich environment. Optical bandgap measurements conducted via photoluminescence (PL) and UV–Vis absorption spectroscopy revealed a bandgap of 2.49 eV (Figure 3b,c), consistent with previously observed values in thin-film materials [23]. These findings confirm the successful synthesis of the semiconducting phase of borophene through the in situ thermal decomposition of KBH_4_. Based on morphological and structural characterizations, the optimal conditions for borophene nanosheet growth were determined to be 120 min of growth at 600 °C.

To further explore the electronic structure and elemental composition, XPS analysis was performed on samples deposited on SiO_2_/Si substrates (Figure 3d). The XPS results indicated a dominant elemental composition of boron, which constituted 67.91% of the sample, as detailed in the elemental composition table. The high-resolution B1s XPS spectrum (Figure 3e) revealed distinct peaks at 187.8 and 188.7 eV, corresponding to strong B-B bonds, consistent with the reported α’-4H-borophene structure [20,24]. High-resolution XPS mapping of C1s further validated these results (Figure 3f).

From a theoretical perspective, alkali metal atoms (Li, Na, K) adsorbed on boron sheets can significantly enhance hydrogen binding energy, thereby stabilizing the borophene structure [25]. The formation of borophene in this study can be attributed to the autocatalytic growth mechanism facilitated by the in situ gas template of potassium (K) generated during the thermal decomposition of KBH_4_. The overall decomposition process of KBH_4_ can be described as follows [26,27,28,29]:

KBH_4_ → K + B + 2H_2_ (1)

**Figure 4:**
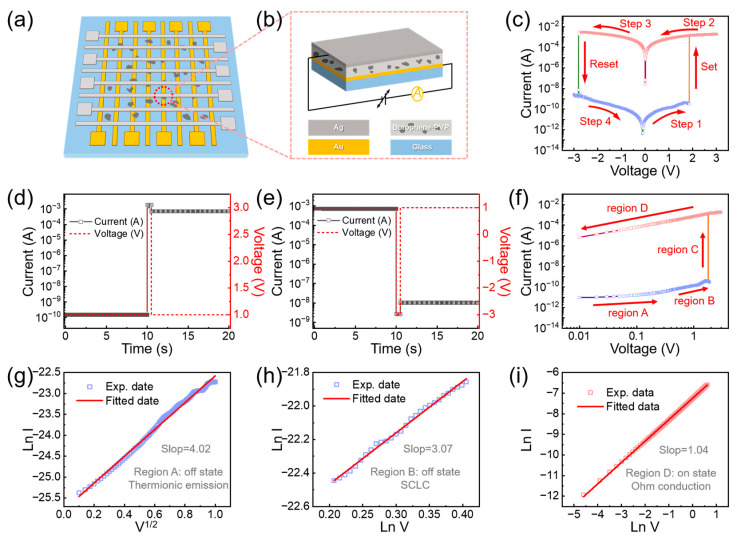
(**a**) Schematic diagram of the Ag/Borophene-PVP/Au/Glass device; (**b**) Cross-sectional diagram of the device; (**c**) Current–voltage (*I*–*V*) curve of the device; (**d**) Depiction of SET operations; (**e**) Depiction of RESET operations; (**f**) *I*–*V* curves of the device during a positive voltage sweep; (**g**) Linear fitting of region A in (**f**); (**h**) Linear fitting of region B in (**f**); (**i**) Linear fitting of region D in (**f**).

Building on the promising electrical properties of borophene nanosheets, a borophene-based non-volatile memory device was designed and fabricated, as shown in Figure 4a. The device structure comprises a silver top electrode, a borophene/PVP active layer, a gold bottom electrode, and a glass substrate (Figure 4b).

During performance testing, the device’s ability to reliably switch between a low-resistance state (LRS, ON state) and a high-resistance state (HRS, OFF state) under voltage scanning was examined. As depicted in Figure 4c, a voltage sweep from 0 to 3 V reveals a sharp current change at approximately 2 V, indicating a transition from the HRS (OFF state) to the LRS (ON state) with a high ON/OFF-current ratio of 10^5^. When the voltage is swept back from 3 V to 0 V, the device maintains the LRS, demonstrating its capability for power-off data retention. In a subsequent voltage sweep from 0 to −3 V, the device undergoes another distinct current change at around −2.7 V, returning to the HRS. The cycle completes as the voltage is swept back from −3 V to 0 V. To further illustrate the switching behavior and rewritable performance, Figure 4d,e presents the response to pulsed voltages of ±3 V. The device consistently performs reliable transitions between ON and OFF states, accurately reflecting its robust switching performance. These results demonstrate the excellent rewritable capabilities and stability of the borophene-based memory device, underscoring its potential for advanced memory storage applications.

To elucidate the operating mechanism of the memory device, a forward voltage scan was performed and divided into four distinct stages, as shown in Figure 4f. These stages correspond to the microelectronic structure of the device during various operating states, as illustrated in Figure S4.

In the initial phase (Region A, OFF state), when the device is subjected to a small forward voltage, the current–voltage relationship exhibits linear behavior, as shown in Figure 4g. The ln(*I*) versus *V*^1/2^ transformation reveals that the device’s conductive mechanism follows the thermionic emission model. The high resistance observed in this phase is attributed to the weak conductivity of the PVP material within the active layer. As the voltage increases (Region B, OFF state), the borophene nanosheets dispersed in the PVP matrix act as charge capture centers. Charges transfer from PVP to borophene due to the lower energy levels of borophene, resulting in charge entrapment within the nanosheets. This process increases the electron concentration and reduces the device’s resistance. The current–voltage relationship in this phase follows a linear curve with a slope of 3.07, consistent with the trap-controlled space charge limited current (SCLC) model (Figure 4h). When the trapped carriers reach a critical concentration, a conductive path forms in the active layer (Region C), leading to a sharp decrease in resistance and a transition from the HRS to the LRS. In the final phase (Region D, ON state), under a positive voltage, the device exhibits stable low-resistance ohmic contact behavior. This behavior is characterized by a linear relationship with a slope of 1.04 in the ln(*I*) versus ln(*V*) plot (Figure 4i). This stable conducting state confirms that the SET process effectively forms a conductive filament, with the trapped charges within the borophene insulated by PVP. This highlights the device’s excellent non-volatile characteristics. When a reverse voltage is applied, the charges in the active layer are neutralized or released, causing the device to transition back to the HRS and completing a full erasure cycle. This process demonstrates the reversible and reliable switching mechanism of the borophene-based memory device. This mechanism is similar to that of our previous work [20].

**Figure 5:**
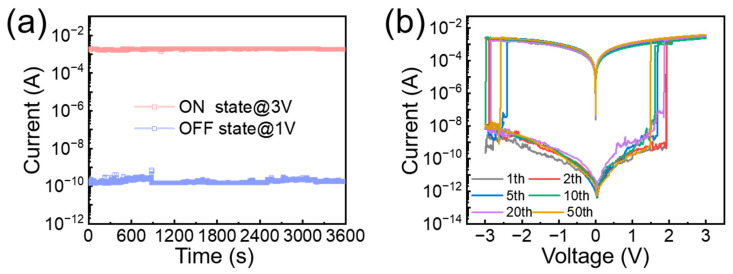
(**a**) Long-term stability test of the device in ON/OFF state; (**b**) *I*–*V* curves of the planar device across the 1st to 50th cycles indicating non-volatility.

Long-term stability and cycling performance of the device were also evaluated. As shown in Figure 5a, the device demonstrated excellent stability, maintaining its OFF state under a 1 V bias and its ON state under a 3 V bias for one hour without significant degradation. Figure 5b further confirms the device’s reliability, showing consistent performance after 50 cycles, indicating its durability and reliable operation as a non-volatile memory device.

This robustness highlights the potential of the device for long-term practical applications. Comparative analysis with other low-dimensional materials, such as black phosphorus quantum dots (BPQDs) [30], functionalized graphene oxide (F-GO) [31], BC_2_N [32], MoS_2_ [33], and α-borophene [34], is summarized in Table S1. Although the SET voltage of the α’-4H-borophene-based device is not among the lowest, its exceptionally high ON/OFF-current ratio makes it highly competitive for applications that demand high-fidelity, high-reliability, and low-power memory devices.

## 4. Conclusions

This study successfully synthesized semiconducting-phase α′-4H-borophene nanosheets via the in situ thermal decomposition of KBH_4_ in a CVD system under a low-pressure, high-purity hydrogen atmosphere. The optimal growth conditions were determined to be 600 °C for 120 min, producing borophene nanosheets with outstanding crystal quality, uniform ultrathin morphology, and excellent crystallinity. The semiconducting properties of the borophene were validated through energy and spectral characterization techniques. Based on this high-quality borophene, a non-volatile memory device was fabricated, featuring a sandwich structure comprising a silver top electrode, a PVP-borophene active layer, and a gold bottom electrode. The device exhibited an impressive ON/OFF-current ratio of approximately 10^5^ and operated at a low voltage of 2 V, benefiting from the superior structural and crystalline properties of borophene. Moreover, the device demonstrated stable performance over extended durations (60 min) and multiple cycles (50 cycles), underscoring its durability and reliability. These results highlight the significant potential of two-dimensional borophene in developing high-performance, high-integration, and high-stability non-volatile memory devices. This study not only confirms the efficacy of the in situ thermal decomposition method for synthesizing high-quality borophene but also provides a robust foundation for its application in next-generation information storage technologies, as well as other electronic or optoelectronic devices.

## Data Availability

Data are contained within the article and Supplementary Materials.

## Supplementary Materials

The following supporting information can be downloaded at: https://www.mdpi.com/article/10.3390/nano15050362/s1, Figure S1: (a) equipment schematic diagram; (b) heating profile; Figure S2: Schematic diagram of the construction of borophene-based non-volatile memory device; Figure S3: SEM images of borophene nanosheets grown for 120 min at different growth temperatures: (a) 550 °C; (b) 575 °C; (c) 625 °C; (d) 650 °C; Figure S4: Working principle of borophene non-volatile memory device; Table S1: Key performance parameters of typical low-dimensional material-based non-volatile memory devices.

## Abbreviations

The following abbreviations are used in this manuscript:

2D	Two-dimensional
CVD	Chemical vapor deposition
TMDs	Transition metal dichalcogenides
BP	Black phosphorus
SEM	Scanning electron microscopy
AFM	Atomic force microscopy
TEM	Transmission electron microscopy
SAED	Selected area electron diffraction
PL	Photoluminescence
XPS	X-ray photoelectron spectroscopy
PVP	Polyvinylpyrrolidone
UV-Vis	Ultraviolet–visible
HRTEM	High-resolution transmission electron microscopy

## References

[1] Novoselov K.S., Geim A.K., Morozov S.V., Jiang D., Zhang Y., Dubonos S.V., Grigorieva I.V., Firsov A.A. (2004). Electric Field Effect in Atomically Thin Carbon Films. Science.

[2] Novoselov K.S., Fal’ko V.I., Colombo L., Gellert P.R., Schwab M.G., Kim K. (2012). A roadmap for graphene. Nature.

[3] Venkata Subbaiah Y.P., Saji K.J., Tiwari A. (2016). Atomically Thin MoS_2_: A Versatile Nongraphene 2D Material. Adv. Funct. Mater..

[4] Li L.K., Yu Y.J., Ye G.J., Ge Q.Q., Ou X.D., Wu H., Feng D.L., Chen X.H., Zhang Y.B. (2014). Black phosphorus field-effect transistors. Nat. Nanotechnol..

[5] Peng B., Zhang H., Shao H., Xu Y., Zhang R., Zhu H. (2016). The electronic, optical, and thermodynamic properties of borophene from first-principles calculations. J. Mater. Chem. C.

[6] Wang Z.Q., Lü T.Y., Wang H.Q., Feng Y.P., Zheng J.C. (2019). Review of borophene and its potential applications. Front. Phys..

[7] Mortazavi B., Silani M., Podryabinkin E.V., Rabczuk T., Zhuang X., Shapeev A.V. (2021). First-Principles Multiscale Modeling of Mechanical Properties in Graphene/Borophene Heterostructures Empowered by Machine-Learning Interatomic Potentials. Adv. Mater..

[8] Tai G., Hu T., Zhou Y., Wang X., Kong J., Zeng T., You Y., Wang Q. (2015). Synthesis of Atomically Thin Boron Films on Copper Foils. Angew. Chem. Int. Ed..

[9] Zhang Z.H., Penev E.S., Yakobson B.I. (2017). Two-dimensional boron: Structures, properties and applications. Chem. Soc. Rev..

[10] Wu X.J., Dai J., Zhao Y., Zhuo Z.W., Yang J.L., Zeng X.C. (2012). Two-Dimensional Boron Monolayer Sheets. Acs Nano.

[11] Ou M., Wang X., Yu L., Liu C., Tao W., Ji X., Mei L. (2021). The Emergence and Evolution of Borophene. Adv. Sci..

[12] Jiao Y., Ma F., Bell J., Bilic A., Du A. (2016). Two-Dimensional Boron Hydride Sheets: High Stability, Massless Dirac Fermions, and Excellent Mechanical Properties. Angew. Chem..

[13] Kistanov A.A., Cai Y., Zhou K., Srikanth N., Dmitriev S.V., Zhang Y.W. (2018). Exploring the charge localization and band gap opening of borophene: A first-principles study. Nanoscale.

[14] Hou C., Tai G., Liu Y., Wu Z., Liang X., Liu X. (2023). Borophene-based materials for energy, sensors and information storage applications. Nano Res. Energy.

[15] Rahman A., Rahman M.T., Chowdhury M.A., Ekram S.B., Uddin M.M.K., Islam M.R., Dong L. (2023). Emerging 2D borophene: Synthesis, characterization, and sensing applications. Sens. Actuators A Phys..

[16] Qi P., Chen Q., Tu D., Yao S., Zhang Y., Wang J., Xie C., Pan C., Peng H. (2020). The potential role of borophene as a radiosensitizer in boron neutron capture therapy (BNCT) and particle therapy (PT). Biomater. Sci..

[17] Duo Y., Xie Z., Wang L., Abbasi N.M., Yang T., Li Z., Hu G., Zhang H. (2021). Borophene-based biomedical applications: Status and future challenges. Coord. Chem. Rev..

[18] Xu Y., Zhang P., Xuan X., Xue M., Zhang Z., Guo W., Yakobson B.I. (2022). Borophane Polymorphs. J. Phys. Chem. Lett..

[19] Xu Y., Xuan X., Yang T., Zhang Z., Li S.D., Guo W. (2022). Quasi-Freestanding Bilayer Borophene on Ag(111). Nano Lett..

[20] Hou C., Tai G., Hao J., Sheng L., Liu B., Wu Z. (2020). Ultrastable Crystalline Semiconducting Hydrogenated Borophene. Angew. Chem. Int. Ed..

[21] Parakhonskiy G., Vtech V., Dubrovinskaia N., Caracas R., Dubrovinsky L. (2013). Raman spectroscopy investigation of alpha boron at elevated pressures and temperatures. Solid State Commun..

[22] Hess N.J., Bowden M.E., Parvanov V.M., Mundy C., Kathmann S.M., Schenter G.K., Autrey T. (2008). Spectroscopic studies of the phase transition in ammonia borane: Raman spectroscopy of single crystal NH_3_BH_3_ as a function of temperature from 88 to 330 K. J. Chem. Phys..

[23] Liang X., Hou C., Wu Z., Wu Z., Tai G. (2023). Multilayer α′-4H-borophene growth on gallium arsenide towards high-performance near-infrared photodetector. Nanotechnology.

[24] Werheit H., Filipov V., Kuhlmann U., Schwarz U., Armbrüster M., Leithe-Jasper A., Tanaka T., Higashi I., Lundström T., Gurin V.N. (2010). Raman effect in icosahedral boron-rich solids. Sci. Technol. Adv. Mater..

[25] Er S., de Wijs G.A., Brocks G. (2009). DFT Study of Planar Boron Sheets: A New Template for Hydrogen Storage. J. Phys. Chem. C.

[26] Xiao X.B., Yu W.Y., Tang B.Y. (2008). First-principles study of a double-cation alkali metal borohydride LiK(BH_4_)_2_. J. Phys. Condens. Matter.

[27] Liu Q., Zhang D., Duan L., Zhang G., Wang L., Cao Y., Qiu Y. (2009). Thermally Decomposable KBH_4_ as an Efficient Electron Injection Material for Organic Light-Emitting Diodes. Jpn. J. Appl. Phys..

[28] Nickels E., Jones M., David W., Johnson S.R., Lowton R., Sommariva M., Edwards P. (2008). Tuning the decomposition temperature in complex hydrides: Synthesis of a mixed alkali metal borohydride. Angew. Chem. Int. Ed..

[29] Orimo S., Nakamori Y., Züttel A. (2004). Material properties of MBH_4_ (M=Li, Na, and K). Mater. Sci. Eng. B..

[30] Zhang X., Xie H., Liu Z., Tan C., Luo Z., Li H., Lin J., Sun L., Chen W., Xu Z. (2015). Black Phosphorus Quantum Dots. Angew. Chem. Int. Ed..

[31] Zhuang X.D., Chen Y., Liu G., Li P.P., Zhu C.X., Kang E.T., Noeh K.G., Zhang B., Zhu J.H., Li Y.X. (2010). Conjugated-Polymer-Functionalized Graphene Oxide: Synthesis and Nonvolatile Rewritable Memory Effect. Adv. Mater..

[32] Zhang P., Hou C., Shao W., Liu R., Wu Z., Tai G. (2023). Crystalline BC_2_N quantum dots. Nano Res..

[33] Liu J., Zeng Z., Cao X., Lu G., Wang L.H., Fan Q.L., Huang W., Zhang H. (2012). Preparation of MoS_2_-Polyvinylpyrrolidone Nanocomposites for Flexible Nonvolatile Rewritable Memory Devices with Reduced Graphene Oxide Electrodes. Small.

[34] Liang X., Hao J., Zhang P., Hou C., Tai G. (2022). Freestanding α-rhombohedral borophene nanosheets: Preparation and memory device application. Nanotechnology.

