# Supplementary material for: In Situ Thermal Decomposition of Potassium Borohydride for Borophene Synthesis and Its Application in a High-Performance Non-Volatile Memory Device
Source: Nanomaterials (Basel). 2025 Feb 26;15(5):362. doi: 10.3390/nano15050362 (PMC11902088; doi:10.3390/nano15050362)
Supplement: Supplementary file 1 [file nanomaterials-15-00362-s001.zip › nanomaterials-3493699-supplementary.pdf]

*Article*

# In-Situ Thermal Decomposition of Potassium Borohydride for Borophene Synthesis and Its Application in High-Performance Non-Volatile Memory Device

**Qian Tian †, Xinchao Liang †, Maoping Xu, Yi Liu, Qilong Wu and Guoan Tai ***

State Key Laboratory of Mechanics and Control for Aerospace Structures, Laboratory of Intelligent Nano Materials and Devices of Ministry of Education, College of Aerospace Engineering, Nanjing University of Aeronautics and Astronautics, Nanjing 210016 (China)

† These authors contributed equally to this work.

* Correspondence: taiguoan@nuaa.edu.cn

## Supporting Information

**Figure S1.** (a) equipment schematic diagram; (b) heating profile.

**Figure S2.** Schematic diagram of the construction of borophene-based non-volatile memory device.

**Figure S3.** SEM images of borophene nanosheets grown for 120 min at different growth temperatures: (a) 550 ℃; (b) 575 ℃; (c) 625 ℃; (d) 650 ℃.

**Figure S4.** Working principle of borophene non-volatile memory device.

**Table S1.** Key performance parameters of typical low-dimensional material-based non-volatile memory devices.

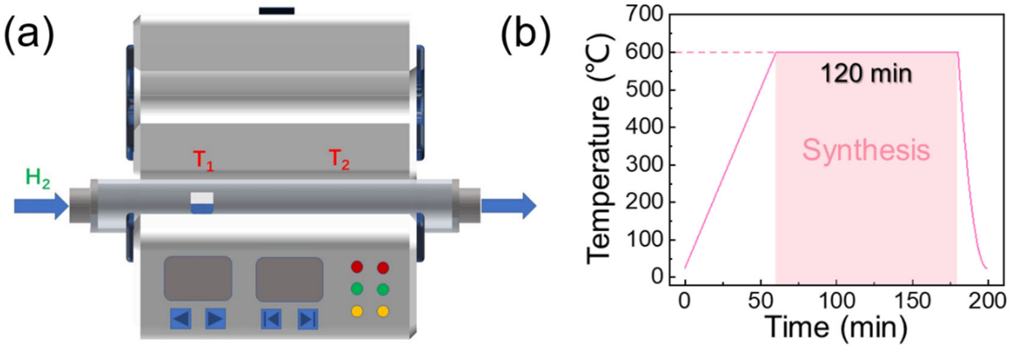


**Figure S1.** (a) equipment schematic diagram; (b) heating profile.

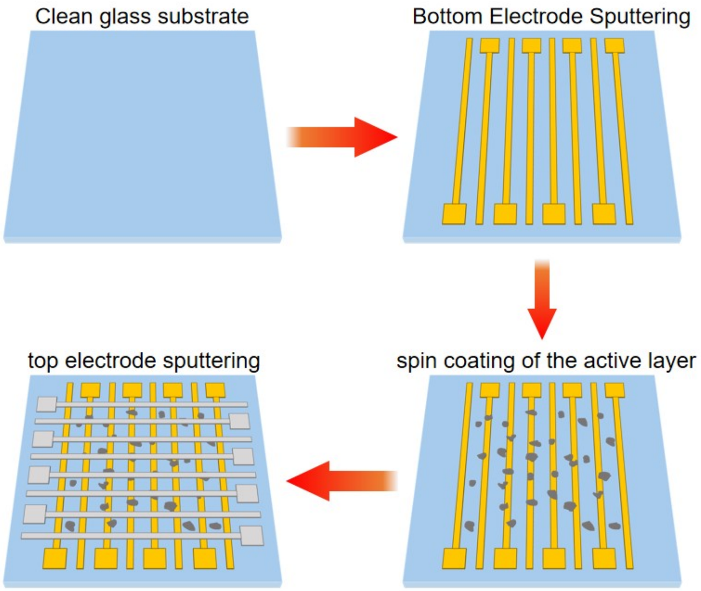


**Figure S2.** Schematic diagram of the construction of borophene-based non-volatile memory device.

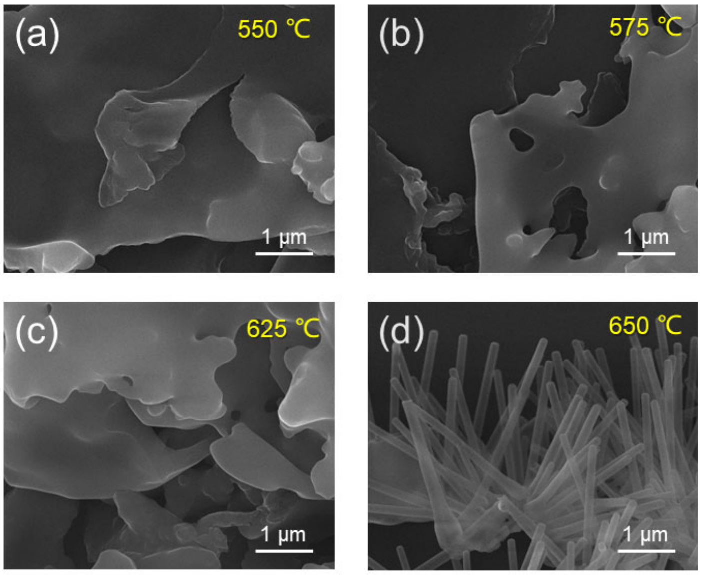


**Figure S3.** SEM images of borophene nanosheets grown for 120 min at different growth temperatures: (a) 550 ℃; (b) 575 ℃; (c) 625 ℃; (d) 650 ℃.

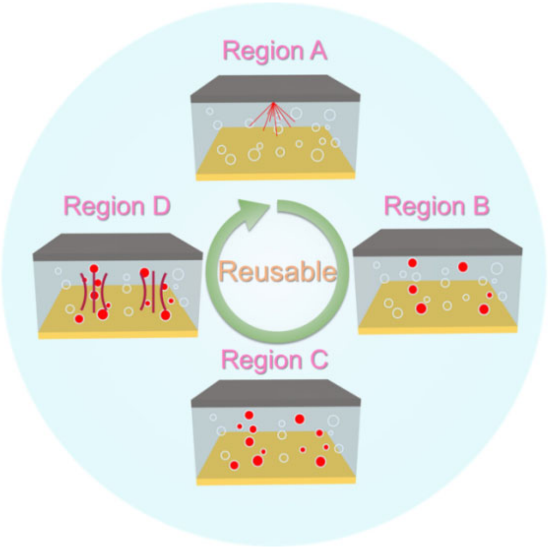


**Figure S4.** Working principle of borophene non-volatile memory device.

**Table S1.** Key performance parameters of typical low-dimensional material-based non-volatile memory devices.

| Material | Set voltage (V) | ON/OFF ratio | Ref. |
| --- | --- | --- | --- |
| BPQD-PVP | 1.2 | ~$6 \times 10^4$ | **Error! Reference source not found.** |
| F-GO-PVP | 1 | ~$10^3$ | **Error! Reference source not found.** |
| $BC_2N$-PVP | 0.74 | ~1740 | **Error! Reference source not found.** |
| $MoS_2$-PVP | 3.5 | ~$10^2$ | **Error! Reference source not found.** |
| $\alpha$-Borophene-PVP | 2 | ~$10^2$ | **Error! Reference source not found.** |
| **$\alpha$'-4H-Borophene-PVP** | **2** | **~$10^5$** | **This work** |
